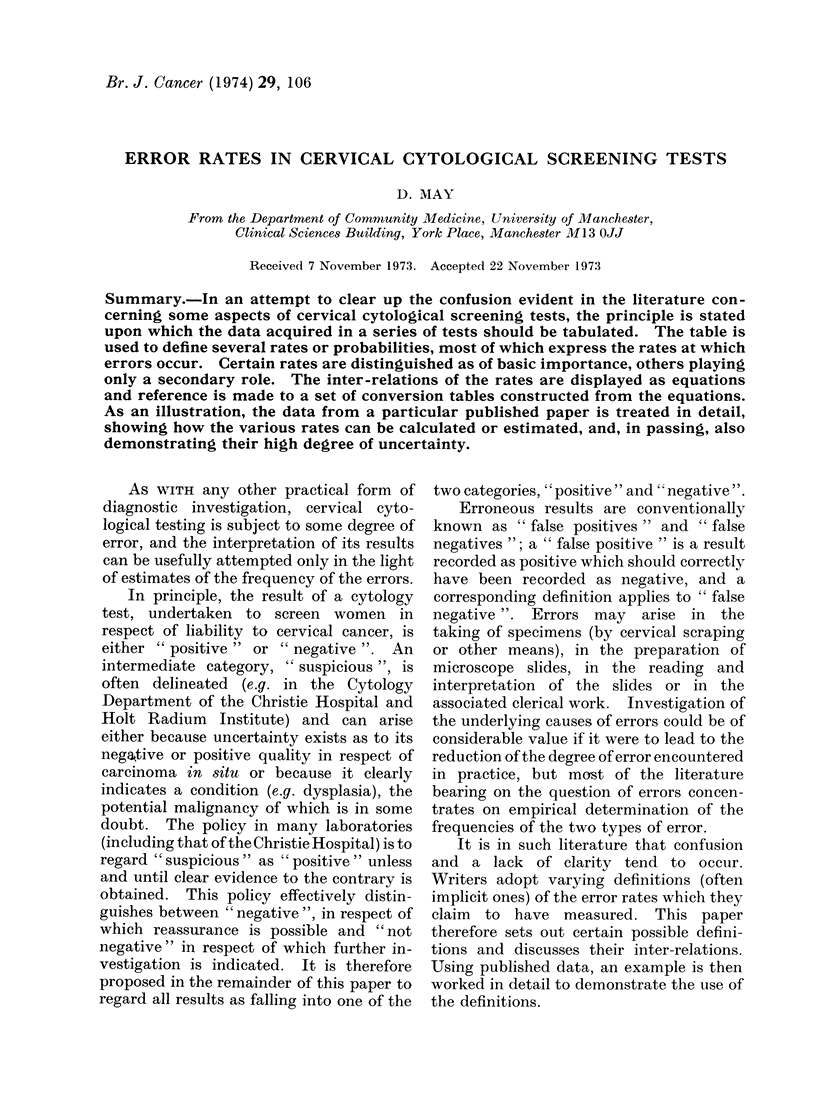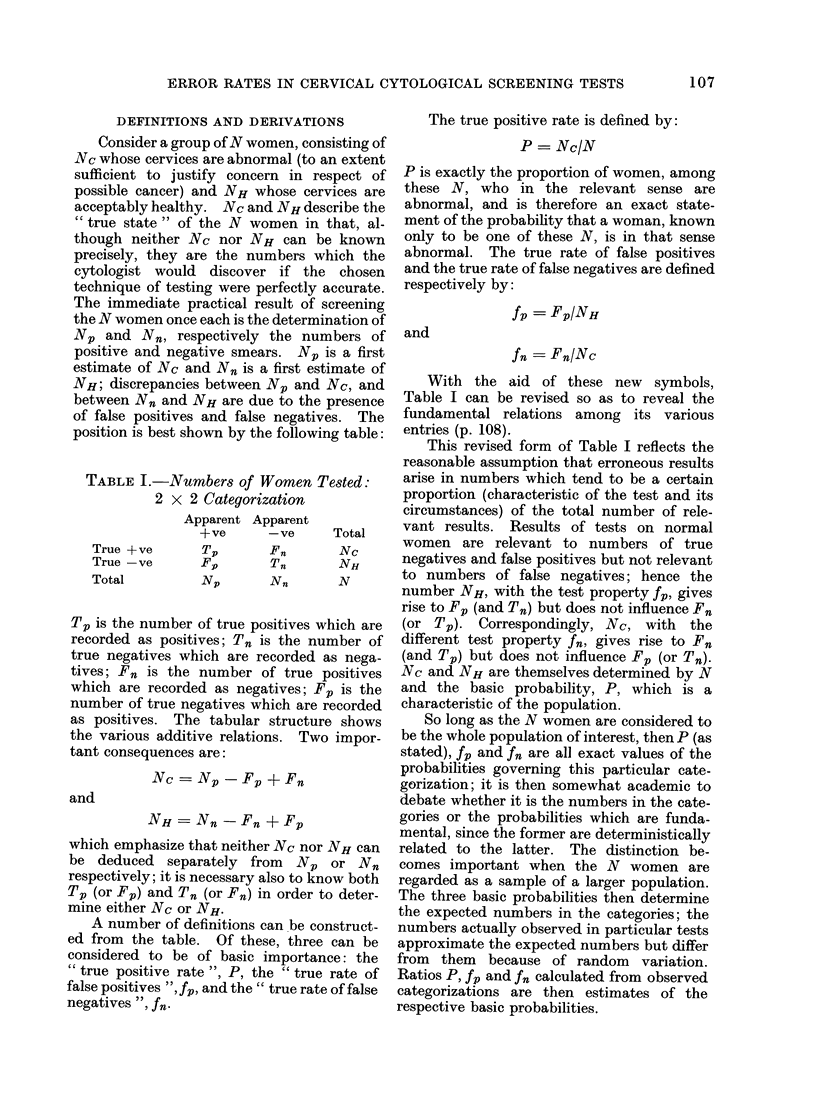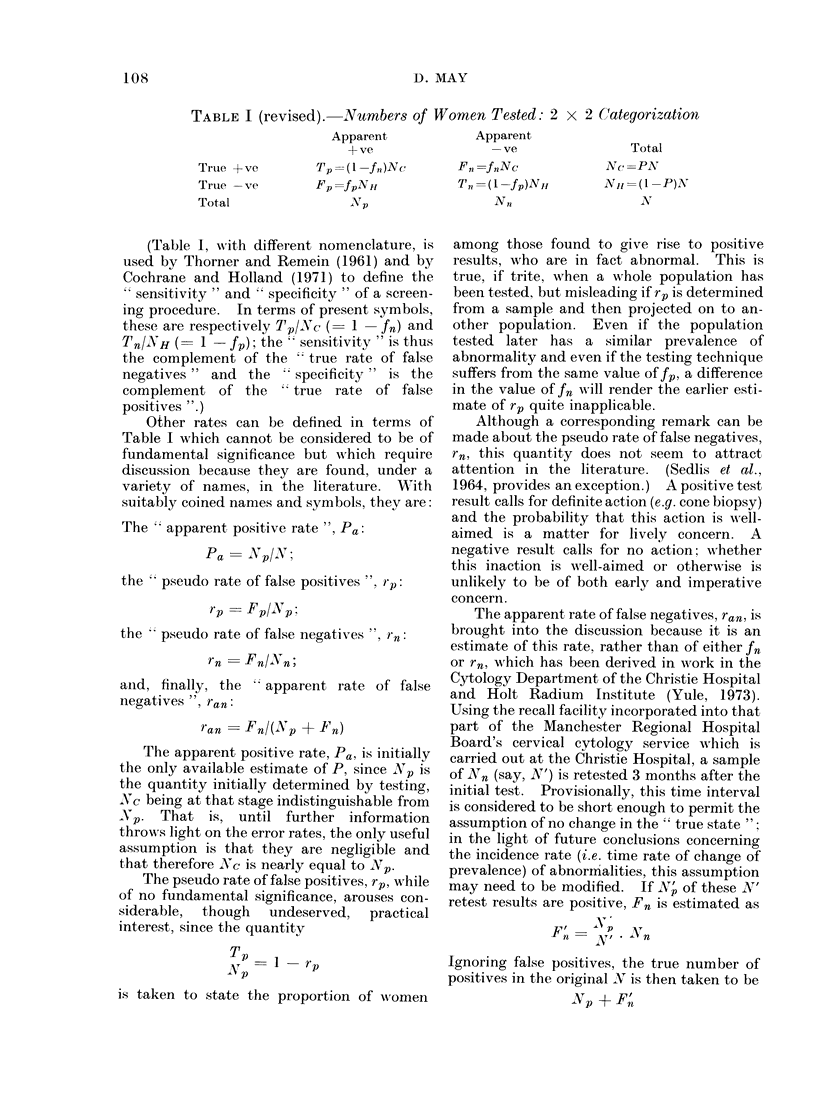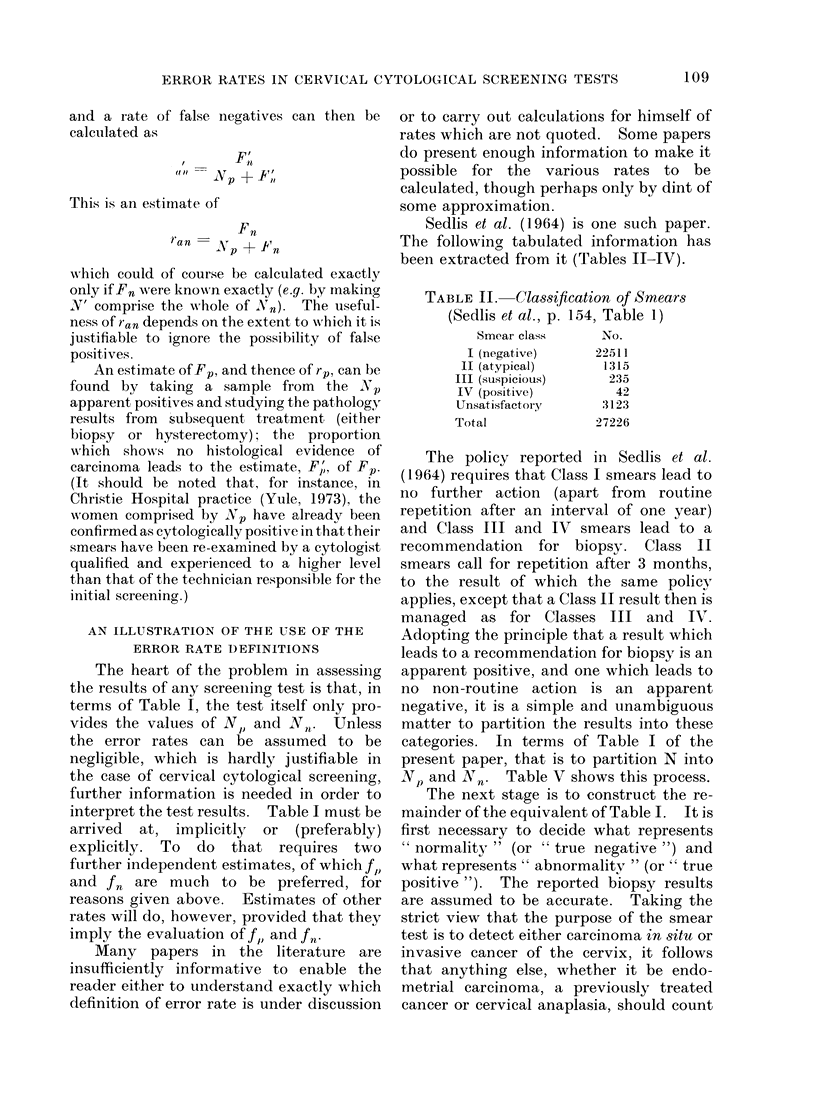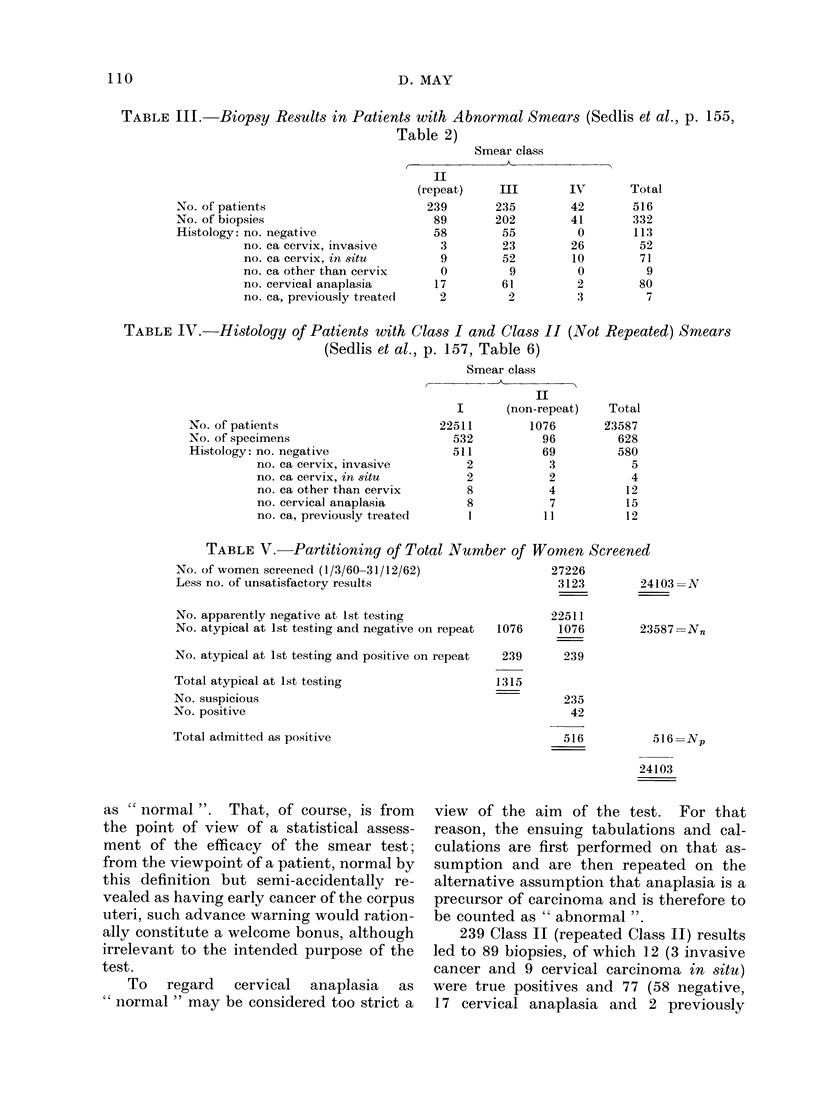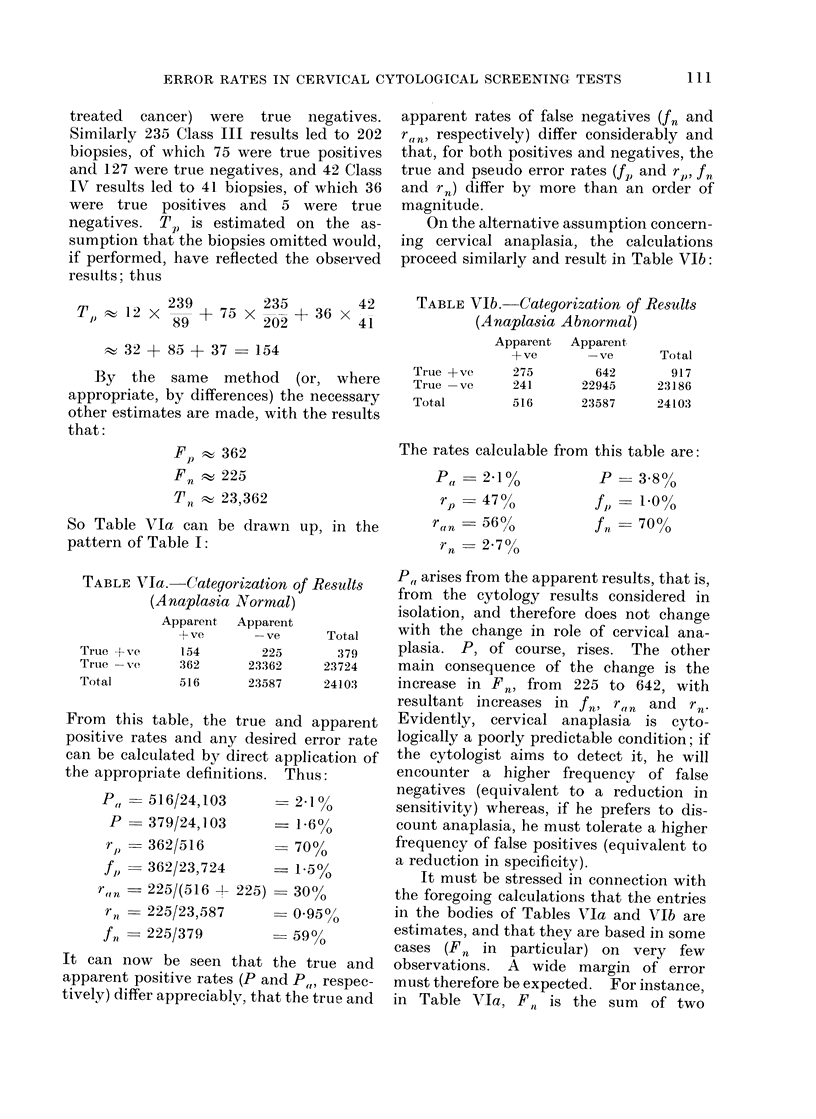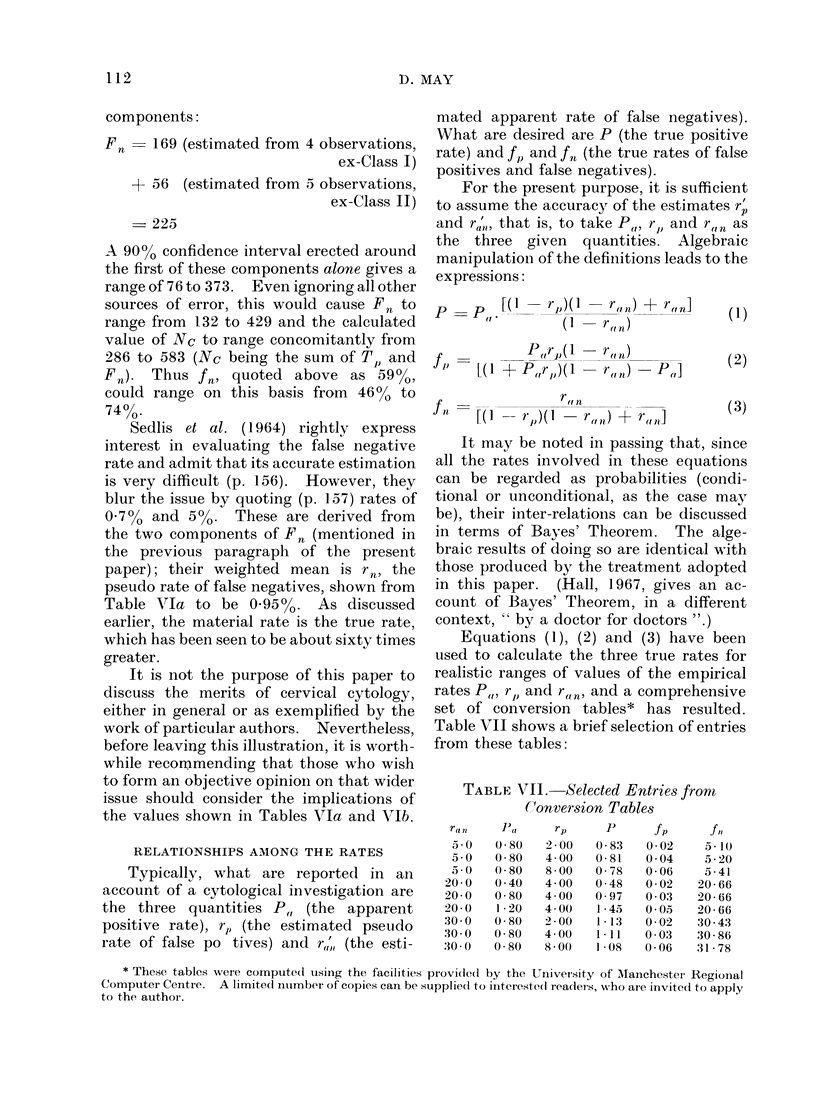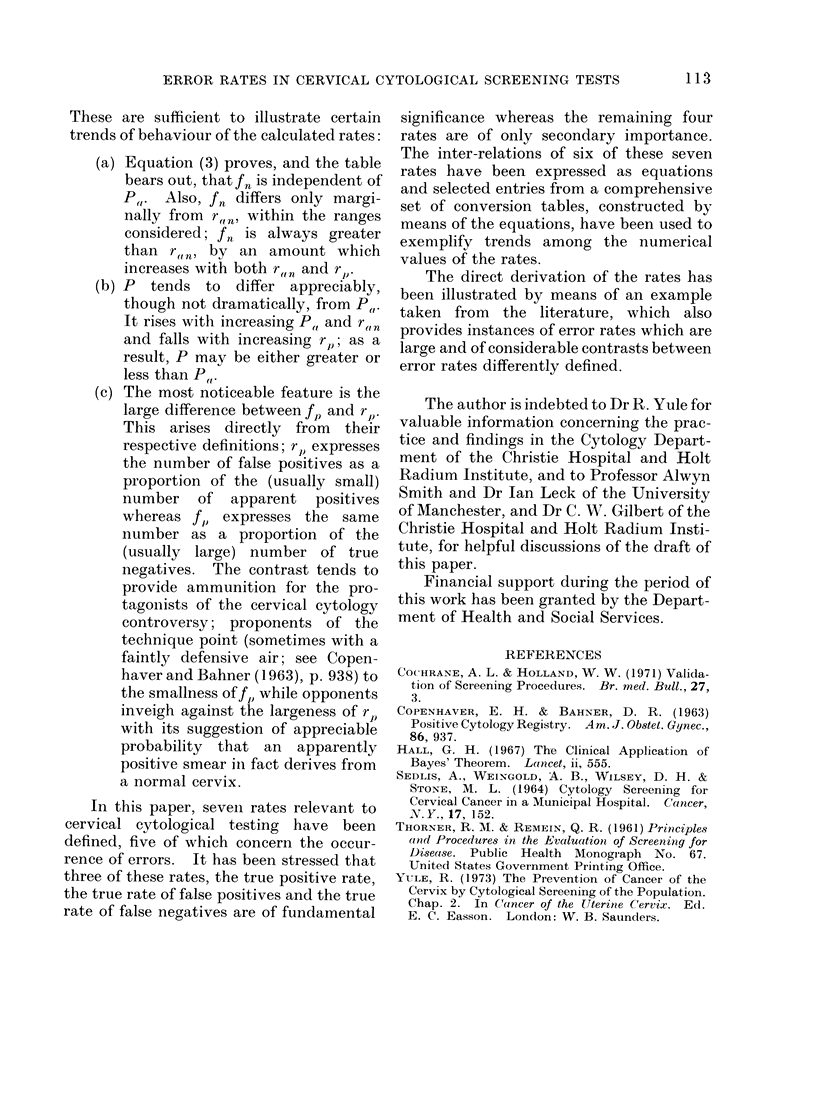# Error Rates in Cervical Cytological Screening Tests

**DOI:** 10.1038/bjc.1974.46

**Published:** 1974-02

**Authors:** D. May

## Abstract

In an attempt to clear up the confusion evident in the literature concerning some aspects of cervical cytological screening tests, the principle is stated upon which the data acquired in a series of tests should be tabulated. The table is used to define several rates or probabilities, most of which express the rates at which errors occur. Certain rates are distinguished as of basic importance, others playing only a secondary role. The inter-relations of the rates are displayed as equations and reference is made to a set of conversion tables constructed from the equations. As an illustration, the data from a particular published paper is treated in detail, showing how the various rates can be calculated or estimated, and, in passing, also demonstrating their high degree of uncertainty.


*Br. J. Cancer* (1974) **29**, 106

### ERROR RATES IN CERVICAL CYTOLOGICAL SCREENING TESTS

D. MAY

*From the Department of Community Medicine, University of Manchester, Clinical Sciences Building, York Place, Manchester M13 0JJ*

Received 7 November 1973. Accepted 22 November 1973

**Summary.—In an attempt to clear up the confusion evident in the literature concerning some aspects of cervical cytological screening tests, the principle is stated upon which the data acquired in a series of tests should be tabulated. The table is used to define several rates or probabilities, most of which express the rates at which errors occur. Certain rates are distinguished as of basic importance, others playing only a secondary role. The inter-relations of the rates are displayed as equations and reference is made to a set of conversion tables constructed from the equations. As an illustration, the data from a particular published paper is treated in detail, showing how the various rates can be calculated or estimated, and, in passing, also demonstrating their high degree of uncertainty.**

As WITH any other practical form of diagnostic investigation, cervical cytological testing is subject to some degree of error, and the interpretation of its results can be usefully attempted only in the light of estimates of the frequency of the errors.

In principle, the result of a cytology test, undertaken to screen women in respect of liability to cervical cancer, is either " positive " or " negative ". An intermediate category, " suspicious ", is often delineated (*e.g.* in the Cytology Department of the Christie Hospital and Holt Radium Institute) and can arise either because uncertainty exists as to its negative or positive quality in respect of carcinoma *in situ* or because it clearly indicates a condition (*e.g.* dysplasia), the potential malignancy of which is in some doubt. The policy in many laboratories (including that of the Christie Hospital) is to regard "suspicious" as "positive" unless and until clear evidence to the contrary is obtained. This policy effectively distinguishes between "negative", in respect of which reassurance is possible and "not negative" in respect of which further investigation is indicated. It is therefore proposed in the remainder of this paper to regard all results as falling into one of the two categories, "positive" and "negative".

Erroneous results are conventionally known as " false positives " and " false negatives "; a " false positive " is a result recorded as positive which should correctly have been recorded as negative, and a corresponding definition applies to " false negative ". Errors may arise in the taking of specimens (by cervical scraping or other means), in the preparation of microscope slides, in the reading and interpretation of the slides or in the associated clerical work. Investigation of the underlying causes of errors could be of considerable value if it were to lead to the reduction of the degree of error encountered in practice, but most of the literature bearing on the question of errors concentrates on empirical determination of the frequencies of the two types of error.

It is in such literature that confusion and a lack of clarity tend to occur. Writers adopt varying definitions (often implicit ones) of the error rates which they claim to have measured. This paper therefore sets out certain possible definitions and discusses their inter-relations. Using published data, an example is then worked in detail to demonstrate the use of the definitions.

### DEFINITIONS AND DERIVATIONS

Consider a group of $N$ women, consisting of $N_C$ whose cervices are abnormal (to an extent sufficient to justify concern in respect of possible cancer) and $N_H$ whose cervices are acceptably healthy. $N_C$ and $N_H$ describe the "true state" of the $N$ women in that, although neither $N_C$ nor $N_H$ can be known precisely, they are the numbers which the cytologist would discover if the chosen technique of testing were perfectly accurate. The immediate practical result of screening the $N$ women once each is the determination of $N_p$ and $N_n$, respectively the numbers of positive and negative smears. $N_p$ is a first estimate of $N_C$ and $N_n$ is a first estimate of $N_H$; discrepancies between $N_p$ and $N_C$, and between $N_n$ and $N_H$ are due to the presence of false positives and false negatives. The position is best shown by the following table:

TABLE I.—*Numbers of Women Tested: 2 × 2 Categorization*

| | Apparent +ve | Apparent −ve | Total |
|---|---|---|---|
| True +ve | $T_p$ | $F_n$ | $N_C$ |
| True −ve | $F_p$ | $T_n$ | $N_H$ |
| Total | $N_p$ | $N_n$ | $N$ |

$T_p$ is the number of true positives which are recorded as positives; $T_n$ is the number of true negatives which are recorded as negatives; $F_n$ is the number of true positives which are recorded as negatives; $F_p$ is the number of true negatives which are recorded as positives. The tabular structure shows the various additive relations. Two important consequences are:

$$N_C = N_p - F_p + F_n$$

and

$$N_H = N_n - F_n + F_p$$

which emphasize that neither $N_C$ nor $N_H$ can be deduced separately from $N_p$ or $N_n$ respectively; it is necessary also to know both $T_p$ (or $F_p$) and $T_n$ (or $F_n$) in order to determine either $N_C$ or $N_H$.

A number of definitions can be constructed from the table. Of these, three can be considered to be of basic importance: the "true positive rate", $P$, the "true rate of false positives", $f_p$, and the "true rate of false negatives", $f_n$.

The true positive rate is defined by:

$$P = N_C/N$$

$P$ is exactly the proportion of women, among these $N$, who in the relevant sense are abnormal, and is therefore an exact statement of the probability that a woman, known only to be one of these $N$, is in that sense abnormal. The true rate of false positives and the true rate of false negatives are defined respectively by:

$$f_p = F_p/N_H$$

and

$$f_n = F_n/N_C$$

With the aid of these new symbols, Table I can be revised so as to reveal the fundamental relations among its various entries (p. 108).

This revised form of Table I reflects the reasonable assumption that erroneous results arise in numbers which tend to be a certain proportion (characteristic of the test and its circumstances) of the total number of relevant results. Results of tests on normal women are relevant to numbers of true negatives and false positives but not relevant to numbers of false negatives; hence the number $N_H$, with the test property $f_p$, gives rise to $F_p$ (and $T_n$) but does not influence $F_n$ (or $T_p$). Correspondingly, $N_C$, with the different test property $f_n$, gives rise to $F_n$ (and $T_p$) but does not influence $F_p$ (or $T_n$). $N_C$ and $N_H$ are themselves determined by $N$ and the basic probability, $P$, which is a characteristic of the population.

So long as the $N$ women are considered to be the whole population of interest, then $P$ (as stated), $f_p$ and $f_n$ are all exact values of the probabilities governing this particular categorization; it is then somewhat academic to debate whether it is the numbers in the categories or the probabilities which are fundamental, since the former are deterministically related to the latter. The distinction becomes important when the $N$ women are regarded as a sample of a larger population. The three basic probabilities then determine the expected numbers in the categories; the numbers actually observed in particular tests approximate the expected numbers but differ from them because of random variation. Ratios $P$, $f_p$ and $f_n$ calculated from observed categorizations are then estimates of the respective basic probabilities.

Table I (revised).—*Numbers of Women Tested: 2 × 2 Categorization*

| | Apparent +ve | Apparent −ve | Total |
|---|---|---|---|
| True +ve | $T_p = (1-f_n)N_C$ | $F_n = f_n N_C$ | $N_C = PN$ |
| True −ve | $F_p = f_p N_H$ | $T_n = (1-f_p)N_H$ | $N_H = (1-P)N$ |
| Total | $N_p$ | $N_n$ | $N$ |

(Table I, with different nomenclature, is used by Thorner and Remein (1961) and by Cochrane and Holland (1971) to define the " sensitivity " and " specificity " of a screening procedure. In terms of present symbols, these are respectively $T_p/N_C$ ($= 1 - f_n$) and $T_n/N_H$ ($= 1 - f_p$); the " sensitivity " is thus the complement of the " true rate of false negatives " and the " specificity " is the complement of the " true rate of false positives ".)

Other rates can be defined in terms of Table I which cannot be considered to be of fundamental significance but which require discussion because they are found, under a variety of names, in the literature. With suitably coined names and symbols, they are:

The " apparent positive rate ", $P_a$:

$$P_a = N_p/N;$$

the " pseudo rate of false positives ", $r_p$:

$$r_p = F_p/N_p;$$

the " pseudo rate of false negatives ", $r_n$:

$$r_n = F_n/N_n;$$

and, finally, the " apparent rate of false negatives ", $r_{an}$:

$$r_{an} = F_n/(N_p + F_n)$$

The apparent positive rate, $P_a$, is initially the only available estimate of $P$, since $N_p$ is the quantity initially determined by testing, $N_C$ being at that stage indistinguishable from $N_p$. That is, until further information throws light on the error rates, the only useful assumption is that they are negligible and that therefore $N_C$ is nearly equal to $N_p$.

The pseudo rate of false positives, $r_p$, while of no fundamental significance, arouses considerable, though undeserved, practical interest, since the quantity

$$\frac{T_p}{N_p} = 1 - r_p$$

is taken to state the proportion of women among those found to give rise to positive results, who are in fact abnormal. This is true, if trite, when a whole population has been tested, but misleading if $r_p$ is determined from a sample and then projected on to another population. Even if the population tested later has a similar prevalence of abnormality and even if the testing technique suffers from the same value of $f_p$, a difference in the value of $f_n$ will render the earlier estimate of $r_p$ quite inapplicable.

Although a corresponding remark can be made about the pseudo rate of false negatives, $r_n$, this quantity does not seem to attract attention in the literature. (Sedlis *et al.*, 1964, provides an exception.) A positive test result calls for definite action (*e.g.* cone biopsy) and the probability that this action is well-aimed is a matter for lively concern. A negative result calls for no action; whether this inaction is well-aimed or otherwise is unlikely to be of both early and imperative concern.

The apparent rate of false negatives, $r_{an}$, is brought into the discussion because it is an estimate of this rate, rather than of either $f_n$ or $r_n$, which has been derived in work in the Cytology Department of the Christie Hospital and Holt Radium Institute (Yule, 1973). Using the recall facility incorporated into that part of the Manchester Regional Hospital Board's cervical cytology service which is carried out at the Christie Hospital, a sample of $N_n$ (say, $N'$) is retested 3 months after the initial test. Provisionally, this time interval is considered to be short enough to permit the assumption of no change in the " true state "; in the light of future conclusions concerning the incidence rate (*i.e.* time rate of change of prevalence) of abnormalities, this assumption may need to be modified. If $N'_p$ of these $N'$ retest results are positive, $F_n$ is estimated as

$$F'_n = \frac{N'_p}{N'} \cdot N_n$$

Ignoring false positives, the true number of positives in the original $N$ is then taken to be

$$N_p + F'_n$$

and a rate of false negatives can then be calculated as

$$r'_{an} = \frac{F'_n}{N_p + F'_n}$$

This is an estimate of

$$r_{an} = \frac{F_n}{N_p + F_n}$$

which could of course be calculated exactly only if $F_n$ were known exactly (*e.g.* by making $N'$ comprise the whole of $N_n$). The usefulness of $r_{an}$ depends on the extent to which it is justifiable to ignore the possibility of false positives.

An estimate of $F_p$, and thence of $r_p$, can be found by taking a sample from the $N_p$ apparent positives and studying the pathology results from subsequent treatment (either biopsy or hysterectomy); the proportion which shows no histological evidence of carcinoma leads to the estimate, $F'_p$, of $F_p$. (It should be noted that, for instance, in Christie Hospital practice (Yule, 1973), the women comprised by $N_p$ have already been confirmed as cytologically positive in that their smears have been re-examined by a cytologist qualified and experienced to a higher level than that of the technician responsible for the initial screening.)

### AN ILLUSTRATION OF THE USE OF THE ERROR RATE DEFINITIONS

The heart of the problem in assessing the results of any screening test is that, in terms of Table I, the test itself only provides the values of $N_p$ and $N_n$. Unless the error rates can be assumed to be negligible, which is hardly justifiable in the case of cervical cytological screening, further information is needed in order to interpret the test results. Table I must be arrived at, implicitly or (preferably) explicitly. To do that requires two further independent estimates, of which $f_p$ and $f_n$ are much to be preferred, for reasons given above. Estimates of other rates will do, however, provided that they imply the evaluation of $f_p$ and $f_n$.

Many papers in the literature are insufficiently informative to enable the reader either to understand exactly which definition of error rate is under discussion or to carry out calculations for himself of rates which are not quoted. Some papers do present enough information to make it possible for the various rates to be calculated, though perhaps only by dint of some approximation.

Sedlis *et al.* (1964) is one such paper. The following tabulated information has been extracted from it (Tables II–IV).

TABLE II.—*Classification of Smears* (Sedlis *et al.*, p. 154, Table 1)

| Smear class | No. |
|---|---|
| I (negative) | 22511 |
| II (atypical) | 1315 |
| III (suspicious) | 235 |
| IV (positive) | 42 |
| Unsatisfactory | 3123 |
| Total | 27226 |

The policy reported in Sedlis *et al.* (1964) requires that Class I smears lead to no further action (apart from routine repetition after an interval of one year) and Class III and IV smears lead to a recommendation for biopsy. Class II smears call for repetition after 3 months, to the result of which the same policy applies, except that a Class II result then is managed as for Classes III and IV. Adopting the principle that a result which leads to a recommendation for biopsy is an apparent positive, and one which leads to no non-routine action is an apparent negative, it is a simple and unambiguous matter to partition the results into these categories. In terms of Table I of the present paper, that is to partition N into $N_p$ and $N_n$. Table V shows this process.

The next stage is to construct the remainder of the equivalent of Table I. It is first necessary to decide what represents "normality" (or "true negative") and what represents "abnormality" (or "true positive"). The reported biopsy results are assumed to be accurate. Taking the strict view that the purpose of the smear test is to detect either carcinoma *in situ* or invasive cancer of the cervix, it follows that anything else, whether it be endometrial carcinoma, a previously treated cancer or cervical anaplasia, should count

TABLE III.—*Biopsy Results in Patients with Abnormal Smears* (Sedlis *et al.*, p. 155, Table 2)

| | Smear class | | | |
|---|---|---|---|---|
| | II (repeat) | III | IV | Total |
| No. of patients | 239 | 235 | 42 | 516 |
| No. of biopsies | 89 | 202 | 41 | 332 |
| Histology: no. negative | 58 | 55 | 0 | 113 |
| no. ca cervix, invasive | 3 | 23 | 26 | 52 |
| no. ca cervix, *in situ* | 9 | 52 | 10 | 71 |
| no. ca other than cervix | 0 | 9 | 0 | 9 |
| no. cervical anaplasia | 17 | 61 | 2 | 80 |
| no. ca, previously treated | 2 | 2 | 3 | 7 |

TABLE IV.—*Histology of Patients with Class I and Class II (Not Repeated) Smears* (Sedlis *et al.*, p. 157, Table 6)

| | Smear class | | |
|---|---|---|---|
| | I | II (non-repeat) | Total |
| No. of patients | 22511 | 1076 | 23587 |
| No. of specimens | 532 | 96 | 628 |
| Histology: no. negative | 511 | 69 | 580 |
| no. ca cervix, invasive | 2 | 3 | 5 |
| no. ca cervix, *in situ* | 2 | 2 | 4 |
| no. ca other than cervix | 8 | 4 | 12 |
| no. cervical anaplasia | 8 | 7 | 15 |
| no. ca, previously treated | 1 | 11 | 12 |

TABLE V.—*Partitioning of Total Number of Women Screened*

| | | | |
|---|---|---|---|
| No. of women screened (1/3/60–31/12/62) | | 27226 | |
| Less no. of unsatisfactory results | | 3123 | $24103 = N$ |
| No. apparently negative at 1st testing | | 22511 | |
| No. atypical at 1st testing and negative on repeat | 1076 | 1076 | $23587 = N_n$ |
| No. atypical at 1st testing and positive on repeat | 239 | 239 | |
| Total atypical at 1st testing | 1315 | | |
| No. suspicious | | 235 | |
| No. positive | | 42 | |
| Total admitted as positive | | 516 | $516 = N_p$ |
| | | | 24103 |

as " normal ". That, of course, is from the point of view of a statistical assessment of the efficacy of the smear test; from the viewpoint of a patient, normal by this definition but semi-accidentally revealed as having early cancer of the corpus uteri, such advance warning would rationally constitute a welcome bonus, although irrelevant to the intended purpose of the test.

To regard cervical anaplasia as " normal " may be considered too strict a view of the aim of the test. For that reason, the ensuing tabulations and calculations are first performed on that assumption and are then repeated on the alternative assumption that anaplasia is a precursor of carcinoma and is therefore to be counted as " abnormal ".

239 Class II (repeated Class II) results led to 89 biopsies, of which 12 (3 invasive cancer and 9 cervical carcinoma *in situ*) were true positives and 77 (58 negative, 17 cervical anaplasia and 2 previously treated cancer) were true negatives. Similarly 235 Class III results led to 202 biopsies, of which 75 were true positives and 127 were true negatives, and 42 Class IV results led to 41 biopsies, of which 36 were true positives and 5 were true negatives. $T_p$ is estimated on the assumption that the biopsies omitted would, if performed, have reflected the observed results; thus

$$T_p \approx 12 \times \frac{239}{89} + 75 \times \frac{235}{202} + 36 \times \frac{42}{41}$$
$$\approx 32 + 85 + 37 = 154$$

By the same method (or, where appropriate, by differences) the necessary other estimates are made, with the results that:

$$F_p \approx 362$$
$$F_n \approx 225$$
$$T_n \approx 23{,}362$$

So Table VI*a* can be drawn up, in the pattern of Table I:

TABLE VI*a*.—*Categorization of Results (Anaplasia Normal)*

| | Apparent +ve | Apparent −ve | Total |
|---|---|---|---|
| True +ve | 154 | 225 | 379 |
| True −ve | 362 | 23362 | 23724 |
| Total | 516 | 23587 | 24103 |

From this table, the true and apparent positive rates and any desired error rate can be calculated by direct application of the appropriate definitions. Thus:

$$P_a = 516/24{,}103 = 2{\cdot}1\%$$
$$P = 379/24{,}103 = 1{\cdot}6\%$$
$$r_p = 362/516 = 70\%$$
$$f_p = 362/23{,}724 = 1{\cdot}5\%$$
$$r_{an} = 225/(516 + 225) = 30\%$$
$$r_n = 225/23{,}587 = 0{\cdot}95\%$$
$$f_n = 225/379 = 59\%$$

It can now be seen that the true and apparent positive rates ($P$ and $P_a$, respectively) differ appreciably, that the true and apparent rates of false negatives ($f_n$ and $r_{an}$, respectively) differ considerably and that, for both positives and negatives, the true and pseudo error rates ($f_p$ and $r_p$, $f_n$ and $r_n$) differ by more than an order of magnitude.

On the alternative assumption concerning cervical anaplasia, the calculations proceed similarly and result in Table VI*b*:

TABLE VI*b*.—*Categorization of Results (Anaplasia Abnormal)*

| | Apparent +ve | Apparent −ve | Total |
|---|---|---|---|
| True +ve | 275 | 642 | 917 |
| True −ve | 241 | 22945 | 23186 |
| Total | 516 | 23587 | 24103 |

The rates calculable from this table are:

$$P_a = 2{\cdot}1\% \qquad P = 3{\cdot}8\%$$

$$r_p = 47\% \qquad f_p = 1{\cdot}0\%$$

$$r_{an} = 56\% \qquad f_n = 70\%$$

$$r_n = 2{\cdot}7\%$$

$P_a$ arises from the apparent results, that is, from the cytology results considered in isolation, and therefore does not change with the change in role of cervical anaplasia. $P$, of course, rises. The other main consequence of the change is the increase in $F_n$, from 225 to 642, with resultant increases in $f_n$, $r_{an}$ and $r_n$. Evidently, cervical anaplasia is cytologically a poorly predictable condition; if the cytologist aims to detect it, he will encounter a higher frequency of false negatives (equivalent to a reduction in sensitivity) whereas, if he prefers to discount anaplasia, he must tolerate a higher frequency of false positives (equivalent to a reduction in specificity).

It must be stressed in connection with the foregoing calculations that the entries in the bodies of Tables VI*a* and VI*b* are estimates, and that they are based in some cases ($F_n$ in particular) on very few observations. A wide margin of error must therefore be expected. For instance, in Table VI*a*, $F_n$ is the sum of two components:

$F_n$ = 169 (estimated from 4 observations, ex-Class I)

+ 56 (estimated from 5 observations, ex-Class II)

= 225

A 90% confidence interval erected around the first of these components *alone* gives a range of 76 to 373. Even ignoring all other sources of error, this would cause $F_n$ to range from 132 to 429 and the calculated value of $N_C$ to range concomitantly from 286 to 583 ($N_C$ being the sum of $T_p$ and $F_n$). Thus $f_n$, quoted above as 59%, could range on this basis from 46% to 74%.

Sedlis *et al.* (1964) rightly express interest in evaluating the false negative rate and admit that its accurate estimation is very difficult (p. 156). However, they blur the issue by quoting (p. 157) rates of 0·7% and 5%. These are derived from the two components of $F_n$ (mentioned in the previous paragraph of the present paper); their weighted mean is $r_n$, the pseudo rate of false negatives, shown from Table VI*a* to be 0·95%. As discussed earlier, the material rate is the true rate, which has been seen to be about sixty times greater.

It is not the purpose of this paper to discuss the merits of cervical cytology, either in general or as exemplified by the work of particular authors. Nevertheless, before leaving this illustration, it is worthwhile recommending that those who wish to form an objective opinion on that wider issue should consider the implications of the values shown in Tables VI*a* and VI*b*.

### RELATIONSHIPS AMONG THE RATES

Typically, what are reported in an account of a cytological investigation are the three quantities $P_a$ (the apparent positive rate), $r_p$ (the estimated pseudo rate of false po tives) and $r'_{an}$ (the estimated apparent rate of false negatives). What are desired are $P$ (the true positive rate) and $f_p$ and $f_n$ (the true rates of false positives and false negatives).

For the present purpose, it is sufficient to assume the accuracy of the estimates $r'_p$ and $r'_{an}$, that is, to take $P_a$, $r_p$ and $r_{an}$ as the three given quantities. Algebraic manipulation of the definitions leads to the expressions:

$$P = P_a \cdot \frac{[(1 - r_p)(1 - r_{an}) + r_{an}]}{(1 - r_{an})} \quad (1)$$

$$f_p = \frac{P_a r_p (1 - r_{an})}{[(1 + P_a r_p)(1 - r_{an}) - P_a]} \quad (2)$$

$$f_n = \frac{r_{an}}{[(1 - r_p)(1 - r_{an}) + r_{an}]} \quad (3)$$

It may be noted in passing that, since all the rates involved in these equations can be regarded as probabilities (conditional or unconditional, as the case may be), their inter-relations can be discussed in terms of Bayes' Theorem. The algebraic results of doing so are identical with those produced by the treatment adopted in this paper. (Hall, 1967, gives an account of Bayes' Theorem, in a different context, " by a doctor for doctors ".)

Equations (1), (2) and (3) have been used to calculate the three true rates for realistic ranges of values of the empirical rates $P_a$, $r_p$ and $r_{an}$, and a comprehensive set of conversion tables* has resulted. Table VII shows a brief selection of entries from these tables:

TABLE VII.—*Selected Entries from Conversion Tables*

| $r_{an}$ | $P_a$ | $r_p$ | $P$ | $f_p$ | $f_n$ |
|---|---|---|---|---|---|
| 5·0 | 0·80 | 2·00 | 0·83 | 0·02 | 5·10 |
| 5·0 | 0·80 | 4·00 | 0·81 | 0·04 | 5·20 |
| 5·0 | 0·80 | 8·00 | 0·78 | 0·06 | 5·41 |
| 20·0 | 0·40 | 4·00 | 0·48 | 0·02 | 20·66 |
| 20·0 | 0·80 | 4·00 | 0·97 | 0·03 | 20·66 |
| 20·0 | 1·20 | 4·00 | 1·45 | 0·05 | 20·66 |
| 30·0 | 0·80 | 2·00 | 1·13 | 0·02 | 30·43 |
| 30·0 | 0·80 | 4·00 | 1·11 | 0·03 | 30·86 |
| 30·0 | 0·80 | 8·00 | 1·08 | 0·06 | 31·78 |

* These tables were computed using the facilities provided by the University of Manchester Regional Computer Centre. A limited number of copies can be supplied to interested readers, who are invited to apply to the author.

These are sufficient to illustrate certain trends of behaviour of the calculated rates:

(a) Equation (3) proves, and the table bears out, that $f_n$ is independent of $P_a$. Also, $f_n$ differs only marginally from $r_{an}$, within the ranges considered; $f_n$ is always greater than $r_{an}$, by an amount which increases with both $r_{an}$ and $r_p$.

(b) $P$ tends to differ appreciably, though not dramatically, from $P_a$. It rises with increasing $P_a$ and $r_{an}$ and falls with increasing $r_p$; as a result, $P$ may be either greater or less than $P_a$.

(c) The most noticeable feature is the large difference between $f_p$ and $r_p$. This arises directly from their respective definitions; $r_p$ expresses the number of false positives as a proportion of the (usually small) number of apparent positives whereas $f_p$ expresses the same number as a proportion of the (usually large) number of true negatives. The contrast tends to provide ammunition for the protagonists of the cervical cytology controversy; proponents of the technique point (sometimes with a faintly defensive air; see Copenhaver and Bahner (1963), p. 938) to the smallness of $f_p$ while opponents inveigh against the largeness of $r_p$ with its suggestion of appreciable probability that an apparently positive smear in fact derives from a normal cervix.

In this paper, seven rates relevant to cervical cytological testing have been defined, five of which concern the occurrence of errors. It has been stressed that three of these rates, the true positive rate, the true rate of false positives and the true rate of false negatives are of fundamental significance whereas the remaining four rates are of only secondary importance. The inter-relations of six of these seven rates have been expressed as equations and selected entries from a comprehensive set of conversion tables, constructed by means of the equations, have been used to exemplify trends among the numerical values of the rates.

The direct derivation of the rates has been illustrated by means of an example taken from the literature, which also provides instances of error rates which are large and of considerable contrasts between error rates differently defined.

The author is indebted to Dr R. Yule for valuable information concerning the practice and findings in the Cytology Department of the Christie Hospital and Holt Radium Institute, and to Professor Alwyn Smith and Dr Ian Leck of the University of Manchester, and Dr C. W. Gilbert of the Christie Hospital and Holt Radium Institute, for helpful discussions of the draft of this paper.

Financial support during the period of this work has been granted by the Department of Health and Social Services.

### REFERENCES

Cochrane, A. L. & Holland, W. W. (1971) Validation of Screening Procedures. *Br. med. Bull.*, **27**, 3.

Copenhaver, E. H. & Bahner, D. R. (1963) Positive Cytology Registry. *Am. J. Obstet. Gynec.*, **86**, 937.

Hall, G. H. (1967) The Clinical Application of Bayes' Theorem. *Lancet*, ii, 555.

Sedlis, A., Weingold, A. B., Wilsey, D. H. & Stone, M. L. (1964) Cytology Screening for Cervical Cancer in a Municipal Hospital. *Cancer, N.Y.*, **17**, 152.

Thorner, R. M. & Remein, Q. R. (1961) *Principles and Procedures in the Evaluation of Screening for Disease.* Public Health Monograph No. 67. United States Government Printing Office.

Yule, R. (1973) The Prevention of Cancer of the Cervix by Cytological Screening of the Population. Chap. 2. In *Cancer of the Uterine Cervix.* Ed. E. C. Easson. London: W. B. Saunders.